# Building a Resilient Scientific Network for COVID-19 and Beyond

**DOI:** 10.1128/mbio.02223-22

**Published:** 2022-09-20

**Authors:** N. Esther Babady, Rachel M. Burckhardt, Florian Krammer, Penny L. Moore, Lynn W. Enquist

**Affiliations:** a Infectious Diseases Service, Department of Medicine, Memorial Sloan Kettering Cancer Center, New York, New York, USA; b Clinical Microbiology Service, Department of Pathology and Laboratory Medicine, Memorial Sloan Kettering Cancer Center, New York, New York, USA; c American Academy of Microbiology, Washington, DC, USA; d Department of Microbiology, Icahn School of Medicine at Mount Sinai, New York, New York, USA; e Department of Pathology, Molecular and Cell Based Medicine, Icahn School of Medicine at Mount Sinai, New York, New York, USA; f Center for Vaccine Research and Pandemic Preparedness (C-VARPP), Icahn School of Medicine at Mount Sinai, New York, New York, USA; g National Institute for Communicable Diseases of the National Health Laboratory Services, Johannesburg, South Africa; h MRC Antibody Immunity Research Unit, School of Pathology, University of the Witwatersrand, Johannesburg, South Africa; i Institute of Infectious Disease and Molecular Medicine, University of Cape Town, Cape Town, South Africa; j Centre for the AIDS Programme of Research in South Africa, University of Kwazulu-Natal, Durban, South Africa; k Department of Molecular Biology, Princeton University, Princeton, New Jersey, USA; GSK Vaccines

**Keywords:** coronavirus, immunology, vaccines

## Abstract

The continued evolution of severe acute respiratory syndrome coronavirus 2 (SARS-CoV-2) necessitates that the global scientific community monitor, assess, and respond to the evolving coronavirus disease (COVID-19) pandemic. But the current reactive approach to emerging variants is ill-suited to address the quickly evolving and ever-changing pandemic. To tackle this challenge, investments in pathogen surveillance, systematic variant characterization, and data infrastructure and sharing across public and private sectors will be critical for planning proactive responses to emerging variants. Additionally, an emphasis on incorporating real-time variant identification in point-of-care diagnostics can help inform patient treatment. Active approaches to understand and identify “immunity gaps” can inform design of future vaccines, therapeutics, and diagnostics that will be more resistant to novel variants. Approaches where the scientific community actively plans for and anticipates changes to infectious diseases will result in a more resilient system, capable of adapting to evolving pathogens quickly and effectively.

## OPINION/HYPOTHESIS

Broad sequencing efforts identified the emergence of more transmissible severe acute respiratory syndrome coronavirus 2 (SARS-CoV-2) variants such as Alpha and Beta at the end of 2020. This signaled a critical need to assess and plan for variants with possibly new pathogenic or transmissibility profiles. Unfortunately, current systems are designed to be reactive—instead of proactive—to these new variants. But the quick pace with which some variants spread across the globe highlighted the need for global collaborative networks that allow for rapid and easy sharing of data among academic, commercial, public health, and government sources to monitor, assay, and respond to new variants.

Since SARS-CoV-2 will continue to evolve, building sustainable and resilient scientific networks for real-time data monitoring and sharing allows lessons learned from the coronavirus disease 2019 (COVID-19) pandemic to inform responses to the emergence of new SARS-CoV-2 variants of concern as well as other global pathogens. These actions are enabled by the correct tools, resources, and infrastructure to allow for global pathogen surveillance, constant monitoring, and proactive responses by the scientific community.

## DESIGNING NETWORKS TO SYSTEMATICALLY ASSESS VARIANTS

Globally, several networks to systematically track variants of concern have been established. These include the COVID-19 Genomics UK Consortium (COG-UK), the WHO Technical Advisory Group on SARS-CoV-2 Virus Evolution (TAG-VE), the Network for Genomics Surveillance in South Africa (NGS-SA), as well as the SARS-CoV-2 Assessment of Viral Evolution (SAVE) program in the United States and many others (1).

Several elements are needed for successful tracking and understanding of SARS-CoV-2 variants. First, surveillance of sequences in various databases (GISAID, GenBank etc.) needs to be established. Available sequences allow scientists to determine the frequency of a certain variant at any point in time in different geographic locations and to assess whether the frequency of the variant is increasing. Structural information and information from serology studies (see below) should be incorporated into surveillance systems that allow rapid identification of concerning mutations as soon as they arise. Of course, in order to perform this work, sequences need to be available first. And while some geographic areas are sampled well, little sequence information is available from others (as described below). Interestingly, the division between countries that perform viral surveillance well and those who provide insufficient sequence information is not necessarily a division between high and low-income countries. In practice, social media channels like Twitter have become increasingly common ways for the rapid delivery of information about new variants, often faster than traditional academic information-sharing pathways.

Another key aspect of successful programs that track variants is the *in vitro* characterization of these virus isolates. While a lot can be predicted from just looking at sequences and mutations, the hypotheses formed by these predictions still need to be tested experimentally. Since neutralizing antibody titers have been established as a correlate of protection, testing if new variants escape from sera of previously infected or vaccinated individuals (or from monoclonal antibodies), and to what degree, needs to be tested (2–5). Various assays, including neutralization assays with live viral isolates, pseudotyped particle entry inhibition assays, as well as receptor-binding domain-angiotensin converting enzyme 2 (RBD-ACE2) interaction inhibition assays can be used for this purpose. Neutralization and pseudotyped particle entry inhibition assays very often deliver highly concordant results (although N-terminal domain [NTD] antibodies seem more potent in neutralization assays with live SARS-CoV-2) (6). However, often it takes longer to obtain virus isolates (which is made more complicated by the bureaucratic hurdles of exporting and importing live viruses) than to synthesize the gene for the spike protein of a variant and use it in a pseudotyped particle entry inhibition assay. Neutralizing antibody titers to the variant of question are then often compared to neutralizing antibody titers of the same serum panel against the ancestral virus. If the drop in neutralization is low (e.g., 2-fold) it is likely that circulating neutralizing antibodies will still protect from a new variant. Higher drops in neutralization (e.g.,15- to 20-fold as observed with Omicron) indicate significantly reduced protection from infection and disease. However, many other viral and immune-factors need to be assessed too, including loss of antibody binding to variant virus spike and/or RBD, loss of CD8^+^ and CD4^+^ T cell reactivity, the influence of viral mutations on fusogenicity, the influence on ACE2 binding affinity, the influence on *in vitro* replication capacity and the influence of mutations in nonspike proteins especially those responsible for replicative efficacy and innate immune evasion. One problem here that has not been solved yet is assay standardization for different assays across laboratories, especially when it comes to variants, because the only available international serum standard was targeted toward the ancestral variant and has also been depleted.

Another important aspect of surveillance programs is the availability to assess pathogenicity and immune escape of new variants in animal models. Several models, including transgenic and wild-type mice, ferrets, and nonhuman primates as well as hamsters have been established. Hamsters seem to replicate pathogenicity seen in humans best although they do not mirror all symptoms and metrics seen in humans, especially not with the Omicron variant and its sublineages (7). Animals can be vaccinated ahead of time and then housed until a new variant emerges. Then they can be challenged with the emerging variant and the ancestral strain and protection can be compared. This analysis complements the *in vitro* data and allows for mechanistic studies that are not possible in humans.

Importantly, data generated *in vitro* and in animal models needs to be supplemented with patient data about “infectiousness,” vaccine breakthrough rates/vaccine effectiveness, incubation time, replicative efficacy in the upper respiratory tract as well as severity data. Differences in incubation time are often overlooked but are important because shorter incubation times allow less time for an anamnestic immune response, which in some cases can protect from disease despite initiation of infection. Integration of these data will allow the community to better assess if a new variant constitutes a serious threat and warrants vaccine updates. Ideally, the pipeline allows associations of single mutations in certain positions with phenotypes, which then feeds back into the surveillance part described above and makes it easier to identify concerning variants just by analyzing their sequence.

Importantly, currently there is no quick regulatory pathway for strain changes for vaccines without clinical trials, but hopefully such pathways will become available soon. In order to maximize the impact of updated vaccines on infection waves with concerning new variants it is imperative to generate data quickly and make decisions about vaccine updates after a very brief period. Updated vaccines that become available months after a new variant has caused a wave are of limited use. Similarly, real-time data for escape from therapeutic monoclonal antibodies needs to be shared rapidly with clinicians to allow selection of the most effective treatment depending on which variant is circulating.

## CONNECTING LABORATORY AND CLINICAL DATA FOR PATIENT AND DIAGNOSTIC NEEDS

The COVID-19 pandemic has shown more than ever before the critical role that diagnostics plays in the management of infectious diseases. A WHO communication from March 16, 2020, stated, “You cannot fight fire blindfolded” to encourage countries to tests for COVID-19 (https://www.who.int/emergencies/diseases/novel-coronavirus-2019/events-as-they-happen). The challenges associated with developing and expanding rapid diagnostic tests during the pandemic have been discussed extensively (8, 9). However, unique to the COVID-19 pandemic, has been the extensive use of next-generation sequencing for relatively rapid genomic surveillance of emerging SARS-CoV-2 variants. These variants have had a marked impact not only on transmission and infectivity but also on the performance of diagnostic tests and the effectiveness of various treatments, including monoclonal antibodies. With that knowledge, the question now is “how do we ensure that clinical laboratories are better prepared to diagnose and manage the next SARS-CoV-2 variant or the next emerging pathogen?”

The current model is a reactive one and the reaction tends to be relatively slow and uncoordinated, whereby every clinical laboratory is left to its own devices to figure out how to prepare. While one size does not fit all, there are a few options that could be considered to increase speed and coordination. First, given that the majority of clinical laboratories will rely on commercial diagnostic tests when those are available, a framework that encourages greater transparency between commercial vendors and clinical laboratories is necessary. Unlike laboratory-developed tests (LDTs), details of commercial tests (e.g., specific genomic target sequence or primers/probes sequences) are not known to clinical laboratories. Therefore, the impact of genomic changes on assay performance cannot be predicted locally and relies heavily on prompt notification by the manufacturers. Such notification does not always occur, and often the first sign that an assay is impacted is observed by the astute clinical microbiologist, as shown recently (10). Amplifying this communication system for all assays more systematically for the many tests used by laboratories would benefit all laboratories using commercial tests. A better system would also be for vendors to communicate more effectively with clinical microbiologists running their assays. Even better would be for commercial vendors to perform frequent experiments that assess the continued performance of their tests against circulating variants (which they probably do) *and* consistently share those data with clinical microbiologists even if the impact is not complete loss of analytical sensitivity or specificity.

A similar approach should be considered with therapeutics as several antivirals, particularly monoclonal antibodies, have lost activity against emerging variants over the course of the pandemic and required clinicians to quickly modify treatment recommendations (11). Given that most laboratories were not able to identify in real time the presence of emerging variants that were resistant to current treatment, the decisions were made at the population level and only when the prevalence of a circulating variant was already high. A better approach would be for clinical laboratories to have the ability to identify variants or key mutations that have a significant impact on treatment rapidly. These assays (when commercially available) are currently limited to identification of known variants when available and not performed in real time but rather only after detection of the SARS-CoV-2 positive sample. This is another opportunity to have a positive impact on patient care, with rapid assays that provide additional variant of concerns information.

There is an urgency associated with diagnosis and treatment of SARS-CoV-2 variants, particularly in immunosuppressed patients. Our current reactive approach is not ideal. The entire genome sequence of the original Wuhan SARS-CoV-2 strain was made publicly available only a few weeks after the initial report of this new syndrome (https://www.who.int/emergencies/diseases/novel-coronavirus-2019/interactive-timeline#event-18). Thus, it stands to reason that a more proactive approach to variants identification could have been developed using modeling and predictive analysis methods to identify hot spots in the genome that could impact testing and therapeutics (12–14). This can still be done, and more efforts should be made to support these types of investigations in order to be better prepared for the next SARS-CoV-2 variant or the next emerging pathogen.

## GLOBAL DATA SHARING FOR PREDICTING IMMUNITY GAPS AND RESPONDING PROACTIVELY

The strengthening of global genomic surveillance systems has proven invaluable in the identification of new SARS-CoV-2 variants. The establishment of the Africa CDC Pathogen Institute for Pathogen Genomics initiative (PGI) is one success story from the pandemic, with next-generation sequencing capacity having expanded dramatically since 2020 (https://ipg.africacdc.org). However, variable genomics capacity continues to result in large regions of the world that are poorly surveyed. These undersequenced regions in some cases overlap high levels of HIV prevalence (https://www.unaids.org/en/resources/documents/2021/2021_unaids_data). As uncontrolled HIV infection is associated with long-term shedding, such populations likely represent a potential source of new SARS-CoV-2 variants of concern (15). Expanding next-generation sequencing capacity yet further remains crucial and may become more challenging as sequencing resources for SARS-CoV-2 sequencing diminish with a global transition to endemicity.

Thus far, our response to the pandemic has been highly reactive, with phenotypic characterization of variants occurring within days or weeks, followed by epidemiological assessments of the spread and clinical severity of new variants. Proactively translating genomics to understand the potential risk of new variants is more difficult. Our understanding of the significance of individual mutations in the spike gene has expanded, informed by laboratory assays of mutants, and modeling of resulting data to enable antigenic cartography (16, 17). We also now have a more detailed understanding of the antibody response to SARS-CoV-2 infection and vaccination at the molecular level, a result of several studies where large numbers of monoclonal antibodies were isolated and characterized (18). This knowledge enables us to evaluate emerging variants at the sequence level fairly rapidly and predict likely immune evasion mutations. However, viral trade-offs in transmissibility and immune evasion mean that mutations need to be considered within context, necessitating continued phenotypic evaluation of new variants.

Leveraging genomics data to define the immunological landscape is becoming increasingly difficult, with most of the world now exhibiting immunity to SARS-CoV-2, either through vaccination, infection, or a combination of both—so-called “hybrid immunity.” Immune profiles likely vary considerably by location and choice of vaccine schedule taken up by a country. For example, much of Africa lags in vaccine coverage, but several studies indicate >90% seroprevalence through high force of infection in sequential waves (19, 20). In contrast, vaccine coverage in the developed world is much higher, though in the era of Omicron, now increasingly followed by infection. Immunological exposure histories will almost certainly shape population immunity, resulting in variable “immunity gaps” where emerging SARS-CoV-2 variants will spread. Different variants trigger slightly different specificities—for example the Beta variant triggered responses with greater cross-reactivity, whereas Omicron (BA.1) infection appears to trigger more variant-specific responses (21, 22). Whether or not this extends to currently circulating sublineages of Omicron remains unknown. Furthermore, a major gap in our understanding of immune responses is a paucity of data on mucosal responses, which are probably more efficiently triggered by infection compared to vaccination (23). Defining the correlation, if any, between systemic and mucosal responses, and ascertaining which better predicts protection from infection is a key gap in the field, with implications for the possible prioritization of intranasal vaccines.

Another key knowledge gap that will enable us to respond more proactively to emerging variants is the lack of any clear correlates of protection from severe disease. While T cell responses and Fc effector functions mediated by binding antibodies against SARS-CoV-2 variants of concern have thus far been remarkably resilient (24, 25), their true contribution to protection from severe disease is correlative at best. To study T cells, quantitative analyses are complicated by the need for stored peripheral blood mononuclear cells and the need to link these laboratory measurements to cases of severe illness, which is becoming increasingly rare, fortunately. Additionally, we will need to continue to assess the possibility of T cell escape. In Omicron, 75 to 80% of T cell responses were preserved, but loss of activity in some individual may suggest the possibility of future escape (24).

Overall, retaining and expanding sequencing capacity should be allied to the strengthening of global laboratory capacity to characterize new variants. Furthermore, while excellent databases are available for the deposition and curation of genomic data, such global databases do not exist for immunological data, and while this would be complicated by the lack of assay standardization mentioned above, these should be prioritized.

## PREPARATION AND PROACTIVE STEPS FOR GLOBAL HEALTH

SARS-CoV-2 has infected millions worldwide, with the long-term consequences on global health and equity unknown. The ever-changing SARS-CoV-2 genomic landscape makes it hard to predict the future of the pandemic, resulting in scientific and public health groups being reactive instead of proactive. The authors suggest the following steps for the scientific community to take to become more proactive in responding to SARS-CoV-2 and other pathogens such as HIV:

- Continue and expand pathogen genomic sequencing for equitable global surveillance, especially in communities with high disease burden that can lead to extended viral evolution and shedding.
- Standardize systematic variant characterization assays *in vitro* (especially neutralization) and in animal models for easier and faster analysis of emerging variants’ impact on diagnostics, therapeutics, and vaccines.
- Establish global communication networks among academic, commercial, public health, and government sources to share data and reagents (including virus isolates) in real time.
- Increase training of clinical microbiologists on next-generation sequencing technology to ensure availability of real-time data on pathogens evolution and the associated impact on the use of diagnostics, vaccines, therapeutics, and patient clinical outcomes.
- Implement point-of-care (POC) tests that identify key genetic mutations associated with reduced therapeutic efficacy to inform patient treatment in real time.
- Analyze the vast genomic and antigenic data of SARS-CoV-2 to identify immunologically important genomic regions, which can help inform design of diagnostics and therapeutics more resistant to emerging variants.

The pandemic provided challenges and opportunities for the scientific community. While vaccine and treatment development occurred at an unprecedented pace, continued viral evolution resulted in immune evasion and therapeutic resistance by emerging variants. Fortunately, we too have the ability to evolve and adapt to the changing pandemic. Proactive monitoring and preparation will result in a system more resilient to pathogen evolution. Global and holistic approaches will be needed. For example, efforts should focus not only on treating those with COVID-19 but on treating people for diseases that lead to compromised immune responses as well. These proactive measures help prevent emergence of new variants arising from long-term infections in immunocompromised individuals and increase the overall health of communities. Together, these actions are the first steps in building a more resilient and connected scientific network to share, analyze, and build upon data that can help us better plan for “what’s next” for COVID-19 and beyond.

## References

[1] DeGrace MM, Ghedin E, Frieman MB, Krammer F, Grifoni A, Alisoltani A, Alter G, Amara RR, Baric RS, Barouch DH, Bloom JD, Bloyet L-M, Bonenfant G, Boon ACM, Boritz EA, Bratt DL, Bricker TL, Brown L, Buchser WJ, Carreño JM, Cohen-Lavi L, Darling TL, Davis-Gardner ME, Dearlove BL, Di H, Dittmann M, Doria-Rose NA, Douek DC, Drosten C, Edara V-V, Ellebedy A, Fabrizio TP, Ferrari G, Fischer WM, Florence WC, Fouchier RAM, Franks J, García-Sastre A, Godzik A, Gonzalez-Reiche AS, Gordon A, Haagmans BL, Halfmann PJ, Ho DD, Holbrook MR, Huang Y, James SL, Jaroszewski L, Jeevan T, Johnson RM, et al. 2022. Defining the risk of SARS-CoV-2 variants on immune protection. Nature 605:640–652. doi:10.1038/s41586-022-04690-5. 35361968 PMC9345323

[2] Goldblatt D, Fiore-Gartland A, Johnson M, Hunt A, Bengt C, Zavadska D, Snipe HD, Brown JS, Workman L, Zar HJ, Montefiori D, Shen X, Dull P, Plotkin S, Siber G, Ambrosino D. 2022. Towards a population-based threshold of protection for COVID-19 vaccines. Vaccine 40:306–315. doi:10.1016/j.vaccine.2021.12.006. 34933765 PMC8673730

[3] Earle KA, Ambrosino DM, Fiore-Gartland A, Goldblatt D, Gilbert PB, Siber GR, Dull P, Plotkin SA. 2021. Evidence for antibody as a protective correlate for COVID-19 vaccines. Vaccine 39:4423–4428. doi:10.1016/j.vaccine.2021.05.063. 34210573 PMC8142841

[4] Khoury DS, Cromer D, Reynaldi A, Schlub TE, Wheatley AK, Juno JA, Subbarao K, Kent SJ, Triccas JA, Davenport MP. 2021. Neutralizing antibody levels are highly predictive of immune protection from symptomatic SARS-CoV-2 infection. Nat Med 27:1205–1211. doi:10.1038/s41591-021-01377-8. 34002089

[5] Gilbert PB, Montefiori DC, McDermott AB, Fong Y, Benkeser D, Deng W, Zhou H, Houchens CR, Martins K, Jayashankar L, Castellino F, Flach B, Lin BC, O'Connell S, McDanal C, Eaton A, Sarzotti-Kelsoe M, Lu Y, Yu C, Borate B, van der Laan LWP, Hejazi NS, Huynh C, Miller J, El Sahly HM, Baden LR, Baron M, De La Cruz L, Gay C, Kalams S, Kelley CF, Andrasik MP, Kublin JG, Corey L, Neuzil KM, Carpp LN, Pajon R, Follmann D, Donis RO, Koup RA, United States Government (USG)/CoVPN Biostatistics Team§. 2022. Immune correlates analysis of the mRNA-1273 COVID-19 vaccine efficacy clinical trial. Science 375:43–50. doi:10.1126/science.abm3425. 34812653 PMC9017870

[6] Sholukh AM, Fiore-Gartland A, Ford ES, Miner MD, Hou YJ, Tse Lv, Kaiser H, Zhu H, Lu J, Madarampalli B, Park A, Lempp FA, st Germain R, Bossard EL, Kee JJ, Diem K, Stuart AB, Rupert PB, Brock C, Buerger M, Doll MK, Randhawa AK, Stamatatos L, Strong RK, McLaughlin C, Huang M-L, Jerome KR, Baric RS, Montefiori D, Corey L. 2021. Evaluation of cell-based and surrogate SARS-CoV-2 neutralization assays. J Clin Microbiol 59. doi:10.1128/JCM.00527-21. PMC8451402 34288726

[7] Halfmann PJ, Iida S, Iwatsuki-Horimoto K, Maemura T, Kiso M, Scheaffer SM, Darling TL, Joshi A, Loeber S, Singh G, Foster SL, Ying B, Case JB, Chong Z, Whitener B, Moliva J, Floyd K, Ujie M, Nakajima N, Ito M, Wright R, Uraki R, Warang P, Gagne M, Li R, Sakai-Tagawa Y, Liu Y, Larson D, Osorio JE, Hernandez-Ortiz JP, Henry AR, Ciuoderis K, Florek KR, Patel M, Odle A, Wong L-YR, Bateman AC, Wang Z, Edara V-V, Chong Z, Franks J, Jeevan T, Fabrizio T, DeBeauchamp J, Kercher L, Seiler P, Gonzalez-Reiche AS, Sordillo EM, Chang LA, van Bakel H, Consortium Mount Sinai Pathogen Surveillance (PSP) study group, et al. 2022. SARS-CoV-2 Omicron virus causes attenuated disease in mice and hamsters. Nature 603:687–692. doi:10.1038/s41586-022-04441-6. 35062015 PMC8942849

[8] Dien Bard J, Babady NE. 2022. The successes and challenges of SARS-CoV-2 molecular testing in the United States. Clin Lab Med 42:147–160. doi:10.1016/j.cll.2022.02.007. 35636819 PMC8901381

[9] Truong TT, Dien Bard J, Butler-Wu SM. 2022. Rapid antigen assays for SARS-CoV-2. Clin Lab Med 42:203–222. doi:10.1016/j.cll.2022.03.001. 35636822 PMC8894733

[10] Rodino KG, Peaper DR, Kelly BJ, Bushman F, Marques A, Adhikari H, Tu ZJ, Marrero Rolon R, Westblade LF, Green DA, Berry GJ, Wu F, Annavajhala MK, Uhlemann A-C, Parikh BA, McMillen T, Jani K, Babady NE, Hahn AM, Koch RT, Grubaugh ND, Rhoads DD, Yale SARS-CoV-2 Genomic Surveillance Initiative. 2022. Partial ORF1ab gene target failure with Omicron BA.2.12.1. J Clin Microbiol 60:e00600-22. doi:10.1128/jcm.00600-22. PMC9199403 35582905

[11] Takashita E, Kinoshita N, Yamayoshi S, Sakai-Tagawa Y, Fujisaki S, Ito M, Iwatsuki-Horimoto K, Halfmann P, Watanabe S, Maeda K, Imai M, Mitsuya H, Ohmagari N, Takeda M, Hasegawa H, Kawaoka Y. 2022. Efficacy of antiviral agents against the SARS-CoV-2 omicron subvariant BA.2. N Engl J Med 386:1475–1477. doi:10.1056/NEJMc2201933. 35263535 PMC8929374

[12] Levinstein Hallak K, Rosset S. 2022. Statistical modeling of SARS-CoV-2 substitution processes: predicting the next variant. Commun Biol 5:1–8. doi:10.1038/s42003-022-03198-y. 35351970 PMC8964801

[13] Beguir K, Skwark MJ, Fu Y, Pierrot T, Lopez Carranza N, Laterre A, Kadri I, Lui BG, Sänger B, Liu Y, Poran A, Muik A, Sahin U. 2021. Early computational detection of potential high risk SARS-CoV-2 variants. bioRxiv. doi:10.1101/2021.12.24.474095. PMC9892295 36774893

[14] Han W, Chen N, Sun S, Gao X. 2022. Predicting the antigenic evolution of SARS-COV-2 with deep learning. bioRxiv. 2022.06.23.497375. 10.1038/s41467-023-39199-6 PMC10261845 37311849

[15] Cele S, Karim F, Lustig G, San JE, Hermanus T, Tegally H, Snyman J, Moyo-Gwete T, Wilkinson E, Bernstein M, Khan K, Hwa S-H, Tilles SW, Singh L, Giandhari J, Mthabela N, Mazibuko M, Ganga Y, Gosnell BI, Karim SSA, Hanekom W, Van Voorhis WC, Ndung'u T, Lessells RJ, Moore PL, Moosa M-YS, de Oliveira T, Sigal A, COMMIT-KZN Team. 2022. SARS-CoV-2 prolonged infection during advanced HIV disease evolves extensive immune escape. Cell Host Microbe 30:154–162. doi:10.1016/j.chom.2022.01.005. 35120605 PMC8758318

[16] Greaney AJ, Starr TN, Gilchuk P, Zost SJ, Binshtein E, Loes AN, Hilton SK, Huddleston J, Eguia R, Crawford KHD, Dingens AS, Nargi RS, Sutton RE, Suryadevara N, Rothlauf PW, Liu Z, Whelan SPJ, Carnahan RH, Crowe JE, Bloom JD. 2021. Complete mapping of mutations to the SARS-CoV-2 spike receptor-binding domain that escape antibody recognition. Cell Host Microbe 29:44–57.e9. doi:10.1016/j.chom.2020.11.007. 33259788 PMC7676316

[17] Wilks SH, Mühlemann B, Shen X, Türeli S, LeGresley EB, Netzl A, Caniza MA, Chacaltana-Huarcaya JN, Corman VM, Daniell X, Datto MB, Dawood FS, Denny TN, Drosten C, Fouchier RAM, Garcia PJ, Halfmann PJ, Jassem A, Jeworowski LM, Jones TC, Kawaoka Y, Krammer F, McDanal C, Pajon R, Simon V, Stockwell MS, Tang H, van Bakel H, Veguilla V, Webby R, Montefiori DC, Smith DJ. 2022. Mapping SARS-CoV-2 antigenic relationships and serological responses. bioRxiv. doi:10.1101/2022.01.28.477987. PMC12145880 37797027

[18] Rogers TF, Zhao F, Huang D, Beutler N, Burns A, He W, Limbo O, Smith C, Song G, Woehl J, Yang L, Abbott RK, Callaghan S, Garcia E, Hurtado J, Parren M, Peng L, Ramirez S, Ricketts J, Ricciardi MJ, Rawlings SA, Wu NC, Yuan M, Smith DM, Nemazee D, Teijaro JR, Voss JE, Wilson IA, Andrabi R, Briney B, Landais E, Sok D, Jardine JG, Burton DR. 2020. Isolation of potent SARS-CoV-2 neutralizing antibodies and protection from disease in a small animal model. Science 369:956–963. doi:10.1126/science.abc7520. 32540903 PMC7299280

[19] Madhi SA, Kwatra G, Myers JE, Jassat W, Dhar N, Mukendi CK, Nana AJ, Blumberg L, Welch R, Ngorima-Mabhena N, Mutevedzi PC. 2022. Population immunity and Covid-19 severity with omicron variant in South Africa. N Engl J Med 386:1314–1326. doi:10.1056/NEJMoa2119658. 35196424 PMC8908853

[20] Bingham J, Cable R, Coleman C, Glatt TN, Grebe E, Mhlanga L, Nyano C, Pieterson N, Swanevelder R, Swarts A, Sykes W, van den Berg K, Vermeulen M, Welte A. 2022. Estimates of prevalence of anti-SARS-CoV-2 antibodies among blood donors in South Africa in March 2022. Res Square. https://www.researchsquare.com/article/rs-1687679/v1.

[21] Moyo-Gwete T, Madzivhandila M, Makhado Z, Ayres F, Mhlanga D, Oosthuysen B, Lambson BE, Kgagudi P, Tegally H, Iranzadeh A, Doolabh D, Tyers L, Chinhoyi LR, Mennen M, Skelem S, Marais G, Wibmer CK, Bhiman JN, Ueckermann V, Rossouw T, Boswell M, de Oliveira T, Williamson C, Burgers WA, Ntusi N, Morris L, Moore PL. 2021. Cross-reactive neutralizing antibody responses elicited by SARS-CoV-2 501Y.V2 (B.1.351). N Engl J Med 384:2161–2163. doi:10.1056/NEJMc2104192. 33826816 PMC8063886

[22] Richardson SI, Madzorera VS, Spencer H, Manamela NP, van der Mescht MA, Lambson BE, Oosthuysen B, Ayres F, Makhado Z, Moyo-Gwete T, Mzindle N, Motlou T, Strydom A, Mendes A, Tegally H, de Beer Z, Roma de Villiers T, Bodenstein A, van den Berg G, Venter M, de Oliviera T, Ueckermann V, Rossouw TM, Boswell MT, Moore PL. 2022. SARS-CoV-2 Omicron triggers cross-reactive neutralization and Fc effector functions in previously vaccinated, but not unvaccinated, individuals. Cell Host Microbe 30:880–886. doi:10.1016/j.chom.2022.03.029. 35436444 PMC8947963

[23] Sano K, Bhavsar D, Singh G, Floda D, Srivastava K, Gleason C, Carreño JM, Simon V, Krammer F, PARIS Study Group. 2021. Efficient mucosal antibody response to SARS-CoV-2 vaccination is induced in previously infected individuals. medRxiv. https://www.medrxiv.org/content/10.1101/2021.12.06.21267352v1.

[24] Keeton R, Tincho MB, Ngomti A, Baguma R, Benede N, Suzuki A, Khan K, Cele S, Bernstein M, Karim F, Madzorera SV, Moyo-Gwete T, Mennen M, Skelem S, Adriaanse M, Mutithu D, Aremu O, Stek C, du Bruyn E, van der Mescht MA, de Beer Z, de Villiers TR, Bodenstein A, van den Berg G, Mendes A, Strydom A, Venter M, Giandhari J, Naidoo Y, Pillay S, Tegally H, Grifoni A, Weiskopf D, Sette A, Wilkinson RJ, de Oliveira T, Bekker L-G, Gray G, Ueckermann V, Rossouw T, Boswell MT, Bhiman JN, Moore PL, Sigal A, Ntusi NAB, Burgers WA, Riou C. 2022. T cell responses to SARS-CoV-2 spike cross-recognize Omicron. Nature 603:488–492. doi:10.1038/s41586-022-04460-3. 35102311 PMC8930768

[25] Richardson SI, Manamela NP, Motsoeneng BM, Kaldine H, Ayres F, Makhado Z, Mennen M, Skelem S, Williams N, Sullivan NJ, Misasi J, Gray GG, Bekker L-G, Ueckermann V, Rossouw TM, Boswell MT, Ntusi NAB, Burgers WA, Moore PL. 2022. SARS-CoV-2 Beta and Delta variants trigger Fc effector function with increased cross-reactivity. Cell Rep Med 3:100510. doi:10.1016/j.xcrm.2022.100510. 35233544 PMC8761540

